# Trend of maternal education in Bangladesh from 2004–2018: Analysis of demographic surveillance data

**DOI:** 10.1371/journal.pone.0255845

**Published:** 2022-01-31

**Authors:** Shathi Das, Fharia Loba, Kamanasis Mozumder, Palash Roy, Jui Das, Sumon Kumar Das

**Affiliations:** 1 St. Gregory’s High School & College, Dhaka, Bangladesh; 2 College of Education, Charles Darwin University, Darwin, Australia; 3 Ministry of Health and Family Welfare, Dhaka, Bangladesh; 4 Kumudini Pharmaceutical Limited, Dhaka, Bangladesh; 5 Centre of Research Excellence in Stillbirth, Mater Research Institute, The University of Queensland, Brisbane, Australia; 6 Menzies School of Health Research, Charles Darwin University, Darwin, Australia; University of Southern Queensland, AUSTRALIA

## Abstract

**Background:**

Maternal education is universally recognised as a major factor in positive societal indicators (health, wellbeing, overall education, etc.) and a country’s growth and economic sustainability, yet the underlying factors contributing to maternal education have not been widely investigated, especially in developing countries.

**Objective:**

This study investigated the 15-year trend of maternal education in Bangladesh (2004–2018) to identify the factors contributing to maternal education.

**Method:**

This study used publicly available cross-data from five consecutive Bangladesh Demographic and Health Surveys (2004, 2007, 2011, 2014 and 2018). Level of maternal education was grouped as ‘no education’, ‘incomplete primary’, ‘complete primary’, ‘incomplete secondary’, ‘complete secondary’ and ‘higher education’ (reference group). The differences in factors/exposure variables suspected to contribute to maternal education were measured for these groups for 2004–2018, and a survey multinomial logistic regression was performed to estimate the explanatory value of these factors.

**Results:**

From 2004–2018, there was a 62% gross reduction of the no education group and a 61% gross increase in the higher education group. A gross increase was also observed for complete secondary (49%), incomplete secondary (39%) and complete primary education (14%). In multivariate analysis, in rural areas, in 2018, the probability of a woman being in the complete primary, incomplete primary or no education groups was increased (adjusted relative risk ratio: 1.21, 1.40 and 1.59), compared to 2004 (0.73, 1.09, 1.12), respectively. From 2004–2018, the factor of no television watching reduced the probability of maternal education levels. Having a husband/partner who had no education increased the probability of a woman’s education level. The probability of all maternal education levels decreased across all wealth index groups.

**Conclusion:**

The data suggest that average maternal education level in Bangladesh increased from 2004–2018. However, an integrated effort is required to improve factors associated with maternal education to both increase maternal education and Bangladesh’s long-term sustainability.

## Introduction

Maternal education is one of the most predominant predictors in public health and social science research [1]. Women’s education level plays a pivotal role in children’s nutrition and cognitive development [2–4], and parents’ maternal education is positively related with their children’s immediate and long-term physical, mental and social wellbeing [5–11]. Well-educated mothers take better care of themselves, contribute to family building and, thus, support proper maintenance of family bonds and social relationships [12]. Maternal education is also an essential factor in improving knowledge, awareness and practice for any public health measures [9]. Women’s education is vital for a country’s economic growth and sustainable development, and policymakers emphasise this in education policy and law [3, 13]. However, a large proportion of young women in many low- and middle-income countries (LMICs) do not have any formal education [14]. Many young women do not complete secondary schooling, even those in developed countries, and even fewer enter tertiary education [15].

The United Nation’s Millennium Development Goals (MDGs) and Sustainable Develop Goals (SDGs) include universal primary education [16]. Bangladesh, despite being a LIMC, is well on track to achieve the MDGs and SDGs targets [17]. There has been a remarkable reduction in the child and maternal mortality rate, improved maternal and child nutrition, prevention of several infectious and non-communicable diseases, and acceleration of the national economy since independence in 1971 [2]. Several recent studies have indicated maternal education as a driving factor for these changes [2, 18]. Yet, despite the importance of maternal education and its widespread use as a risk indicator, there is a lack of research on the factors contributing to maternal education [19]. This study examined the 15-year trend of maternal education in Bangladesh (2004–2018) to identify the factors contributing to maternal education.

## Methods

### Bangladesh Demographic and Health Survey

The Bangladesh Demographic and Health Survey (BDHS) is a cross-sectional, population-representative survey conducted every three to four years since 1993. The survey uses a two-stage stratified sampling design (primary sampling units are defined as per census enumeration areas and households are then randomly selected from these primary sampling units), with stratification by urban and rural areas within each division of the country. The BDHS has used almost identical questionnaires and sampling schemes across the years, which allows for comparison of maternal education levels across various demographics over time. Detailed descriptions of the BDHS sampling and survey method have previously been undertaken [20, 21]. All the survey data (secondary data) are publicly available [22], thus ethical approval was not required for this study. This study used five BDHS datasets from 2004–2018, comprising a total of 76,841 individuals extracted from the ‘BDIR’ data files for the 2004 (N = 11,417), 2007 (N = 10,939), 2011 (N = 17,808), 2014 (N = 17,827) and 2018 (N = 18,850) surveys, after excluding the non-responsive information for the selected variables. Within selected households, every married woman aged 13–49 years were interviewed and asked questions about a wide range of topics (age, education, media exposure, household assets, partner’s education, etc.).

### Primary outcome and explanatory factors/exposure variables of interest

Level of maternal education was the primary outcome, categorised as ‘no education’, ‘incomplete primary’, ‘complete primary’, ‘incomplete secondary’, ‘complete secondary’ and ‘higher education’ (reference group).

The study considered several factors/exposure variables:

- Residency: urban area (reference group) or rural area
- Wealth index (household pre-estimated): richest (reference group), richer, middle, poorer, poorest
- Media exposure (listening to radio, watching television and reading newspapers): at least once a week (reference group), less than once a week and not at all
- Husband’s education level: no education, incomplete primary, complete primary, incomplete secondary, complete secondary and higher education (reference group)
- Religion: Islam (reference group) and non-Islam (note: around 90% of the study’s participants were Muslim, which reflects the religious demographics of Bangladesh)
- Maternal age: ≤18 years and ≥19 years (reference group) (note: age of graduating secondary school was assumed to be 18 years).

### Statistical analysis

This study used descriptive and multivariable analysis to determine the change in women’s education over time and compared estimates across survey years. Change in proportion of women’s education groups between 2004 and 2018 was estimated by:

$$\left(\frac{T1–T2}{T1}\right)\times 100$$

where T1 is Time Point 1 and T2 is Time Point 2.

All maternal education level group proportions were estimated and calculated based on the BDHS design. Individual survey sample weight was used to perform survey multinomial logistic regression (‘suy:mlogit’) to estimate the relative risk ratios (odds ratio) and their 95% confidence intervals for each explanatory factor.

All explanatory factors were included in the multivariate analysis except reading newspapers (because people cannot read unless they are literate/possess a certain level of education). All statistical analyses were conducted using STATA version 15 (Stata Corp, College Station, TX, USA).

## Results

**Fig 1:**
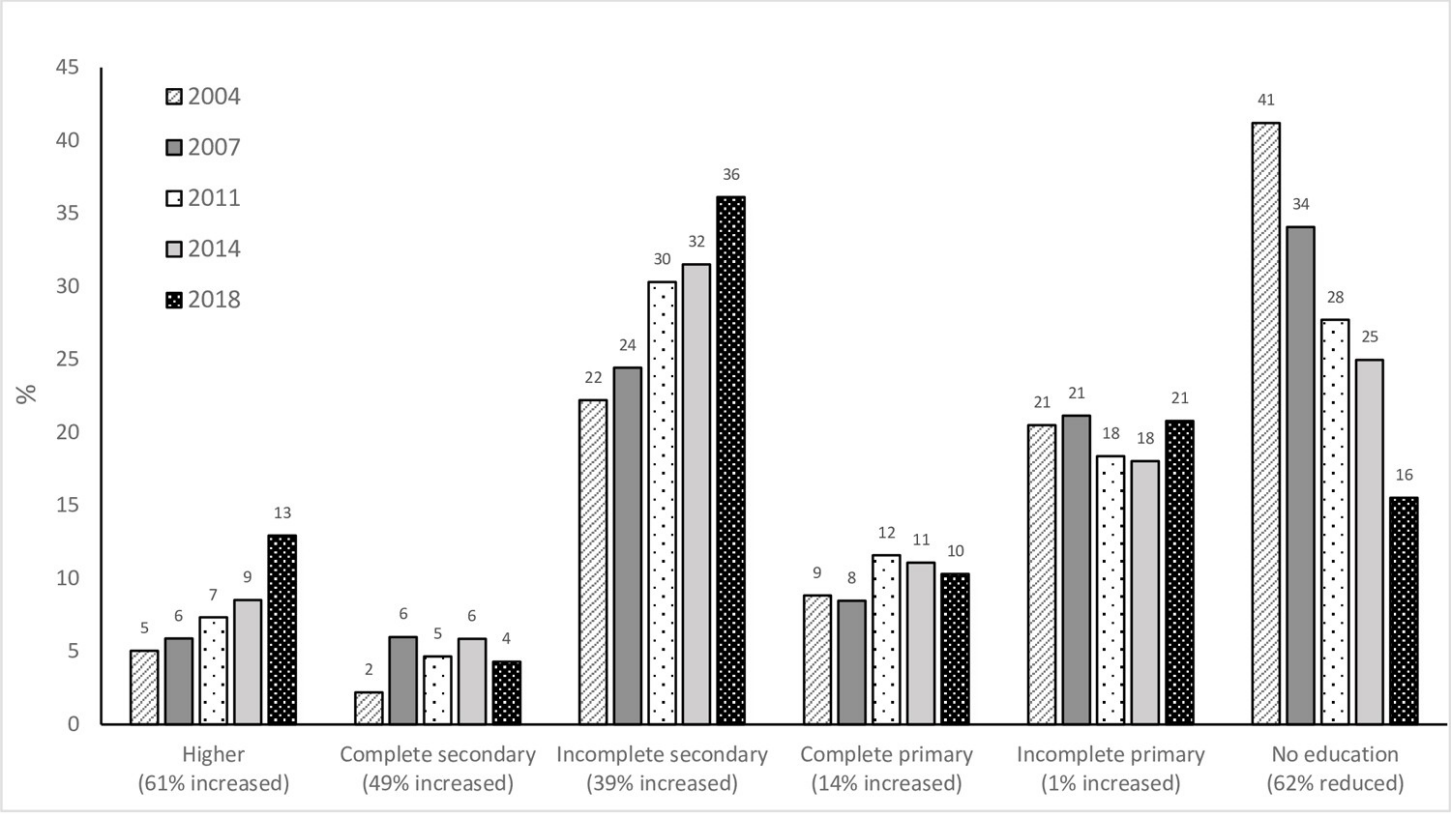
Overall general trends in proportion^1^ of maternal education level groups from 2004–2018.

Fig 1 shows the overall general trends in the proportion of maternal education groups. In 2004, 41% of mothers had no education, which had reduced by 62% by 2018. Only 5% of women had higher education in 2004, which had increased by 61% by 2018. From 2004–2018, the complete secondary, incomplete secondary and complete primary education groups increased by 49%, 39% and 14%, respectively. The proportion of mothers with incomplete primary education remained high and saw a 1% increase from 2004–2018.

From 2004–2018, the proportion of higher education level increased by 24% in urban areas and decreased by 14% in rural areas (Table 1). Similarly, the proportion of women with no education reduced by 3% in urban areas and increased by 8% in rural areas. Non-Muslims saw virtually no change in the proportions of maternal education levels. Maternal education levels decreased among the richest and richer wealth index groups, although maternal education levels increased among mothers in the poorer and poorest wealth index groups (Table 1). Media exposure (listening to radio, watching television and reading newspapers) reduced across all maternal education levels. The proportion of higher educated mothers who had higher educated husbands decreased and the proportion of uneducated husbands decreased (Table 1).

**Table 1:** Change in proportion^1^ of different exposures among different education level groups between 2004 and 2018.

Exposure variables	Higher	Complete secondary	Incomplete secondary	Complete primary	Incomplete primary	No education
Area						
Urban	24.22	21.14	6.07	2.37	0.39	2.88
Rural	13.50	16.74	9.81	5.31	0.86	7.50
Religion						
Islam	3.08	7.53	2.39	0.10	1.93	0.32
Non-Islam	17.39	38.70	16.80	1.04	17.08	2.89
Wealth index						
Richest	25.79	33.05	42.76	49.43	41.17	33.71
Richer	45.34	17.18	14.02	25.97	13.84	4.92
Middle	100.33	90.84	19.27	2.06	11.12	5.08
Poorer	261.64	137.84	64.93	48.09	10.42	8.16
Poorest	910.34	803.03	210.00	127.27	50.74	6.85
Frequency of listening to radio						
At least once a week	86.49	91.29	95.40	97.37	96.78	98.49
Less than once a week	28.21	67.37	73.81	88.20	92.88	96.90
Not at all	83.30	128.48	115.88	99.10	80.01	50.99
Frequency of watching television						
At least once a week	7.99	13.02	7.55	10.89	4.84	13.20
Less than once a week	72.70	31.33	3.88	9.45	2.10	8.08
Not at all	39.06	54.32	16.96	17.88	4.04	5.81
Frequency of reading newspaper						
At least once a week	62.90	79.11	87.12	91.84	92.75	100.00
Less than once a week	2.13	57.89	70.00	87.85	79.00	42.86
Not at all	153.21	170.24	48.33	23.11	3.29	0.02
Husband/partner’s educational attainment						
Higher	14.05	33.44	28.49	45.74	35.66	30.11
Complete secondary	48.68	15.72	23.66	42.29	31.13	26.71
Incomplete secondary	79.41	100.45	2.82	4.74	20.87	9.85
Complete primary	109.66	272.86	59.94	4.30	26.55	9.91
Incomplete primary	144.44	208.15	47.62	49.62	20.88	26.42
No education	41.38	18.05	22.79	6.35	4.88	5.27

Shaded estimates are decreased over time (2004–2018) otherwise increased.

^1^ Unweighted proportion.

Detail data presented in S1 Table (corresponding ratios also presented).

Tables 2 and 3 present the detailed adjusted associations of all variables in 2004 and 2018, respectively. In 2004, rural women were 2% (insignificant) and 26% (significant) less likely to be in the complete secondary, incomplete secondary and complete primary education groups (Table 2). In 2018, rural women were 6% (insignificant) and 3% (insignificant) less likely to be in the complete secondary and incomplete secondary groups, but 21% (significant), 40% (significant) and 59% (significant) more likely to be in the primary, incomplete primary and no education groups (Table 3). The probability of complete secondary education among non-Muslim women decreased in 2018 compared to 2004 (1.02 to 0.67) (Tables 2 and 3).

**Table 2:** Multivariable analysis^1^ of maternal education level and different factors in 2004.

	Complete secondary	Incomplete secondary	Complete primary	Incomplete primary	No education
Age					
19 years and above	Ref.	Ref.	Ref.	Ref.	Ref.
18 years and less	1.82 (0.94–3.51)	3.65 (2.25–5.90)	1.51 (0.90–2.53)	1.01 (0.61–1.68)	0.29 (0.17–0.50)
Area					
Urban	Ref.	Ref.	Ref.	Ref.	Ref.
Rural	0.98 (0.69–1.38)	0.74 (0.58–0.95)	0.73 (0.55–0.98)	1.09 (0.83–1.43)	1.21 (0.92–1.58)
Religion					
Islam	Ref.	Ref.	Ref.	Ref.	Ref.
Non-Islam	1.02 (0.65–1.60)	0.83 (0.60–1.15)	0.51 (0.34–0.76)	0.65 (0.45–0.95)	0.74 (0.51–1.07)
Wealth index					
Richest	Ref.	Ref.	Ref.	Ref.	Ref.
Richer	1.47 (0.96–2.27)	1.63 (1.20–2.22)	2.29 (1.60–3.28)	2.53 (1.80–3.58)	2.60 (1.85–3.64)
Middle	1.08 (0.58–2.01)	1.86 (1.20–2.88)	2.16 (1.34–3.49)	3.88 (2.44–6.17)	3.96 (2.50–6.29)
Poorer	2.16 (0.86–5.40)	2.63 (1.31–5.30)	3.55 (1.70–7.42)	7.44 (3.65–15.19)	8.54 (4.19–17.41)
Poorest	1.16 (0.16–8.71)	2.42 (0.56–10.43)	4.57 (1.04–20.10)	12.11 (2.79–52.57)	18.00 (4.14–78.28)
Frequency of listening to radio					
At least once a week	Ref.	Ref.	Ref.	Ref.	Ref.
Less than once a week	0.85 (0.51–1.41)	1.14 (0.79–1.63)	1.38 (0.92–2.09)	1.22 (0.82–1.80)	1.34 (0.90–1.98)
Not at all	0.90 (0.63–1.28)	1.13 (0.88–1.44)	1.31 (0.99–1.74)	1.32 (1.01–1.73)	1.81 (1.38–2.36)
Frequency of watching television					
At least once a week	Ref.	Ref.	Ref.	Ref.	Ref.
Less than once a week	1.34 (0.63–2.86)	1.45 (0.83–2.54)	2.04 (1.13–3.68)	1.66 (0.93–2.96)	1.94 (1.08–3.47)
Not at all	1.31 (0.80–2.12)	1.71 (1.22–2.39)	2.28 (1.57–3.32)	2.68 (1.87–3.82)	3.72 (2.61–5.30)
Husband/partner’s educational attainment					
Higher	Ref.	Ref.	Ref.	Ref.	Ref.
Complete secondary	6.03 (3.61–10.06)	6.88 (4.44–10.64)	12.52 (7.27–21.58)	14.45 (8.27–25.24)	23.38 (12.96–42.17)
Incomplete secondary	2.45 (1.50–4.01)	13.59 (9.79–18.86)	26.14 (17.28–39.53)	51.92 (34.00–79.27)	80.60 (50.72–128.09)
Complete primary	1.25 (0.43–3.68)	14.05 (7.05–27.97)	76.12 (36.78–157.56)	86.30 (41.41–179.82)	210.41 (99.12–446.65)
Incomplete primary	4.76 (1.29–17.57)	45.46 (18.13–114.00)	144.45 (55.50–375.97)	506.68 (195.53–1312.95)	924.69 (349.86–2443.94)
No education	1.81 (0.45–7.32)	35.13 (13.96–88.41)	157.21 (60.45–408.89)	479.12 (184.23–1246.01)	(889.47–6254.23)

^1^Survey multinomial regression where survey weight for 2004 was used to estimate relative risk ratio and 95% Confidence Interval.

**Table 3:** Multivariable analysis^1^ of maternal education level and different factors in 2018.

	Complete secondary	Incomplete secondary	Complete primary	Incomplete primary	No education
Age					
19 years and above	Ref.	Ref.	Ref.	Ref.	Ref.
18 years and less	1.47 (1.06–2.04)	1.48 (1.17–1.87)	0.80 (0.58–1.09)	0.48 (0.35–0.65)	0.14 (0.09–0.24)
Area					
Urban	Ref.	Ref.	Ref.	Ref.	Ref.
Rural	0.94 (0.77–1.15)	0.97 (0.85–1.12)	1.21 (1.01–1.45)	1.40 (1.19–1.65)	1.59 (1.32–1.90)
Religion					
Islam	Ref.	Ref.	Ref.	Ref.	Ref.
Non-Islam	0.67 (0.50–0.91)	0.73 (0.60–0.88)	0.60 (0.46–0.77)	0.61 (0.49–0.77)	0.78 (0.61–1.01)
Wealth index					
Richest	Ref.	Ref.	Ref.	Ref.	Ref.
Richer	1.12 (0.88–1.42)	1.48 (1.26–1.73)	1.85 (1.47–2.34)	2.08 (1.68–2.58)	1.98 (1.53–2.56)
Middle	1.07 (0.79–1.44)	2.12 (1.75–2.58)	2.47 (1.90–3.22)	3.24 (2.54–4.14)	2.91 (2.20–3.87)
Poorer	0.98 (0.68–1.42)	2.41 (1.87–3.11)	3.40 (2.49–4.64)	4.86 (3.62–6.52)	4.30 (3.10–5.96)
Poorest	0.91 (0.57–1.46)	2.49 (1.81–3.43)	3.52 (2.43–5.12)	6.05 (4.25–8.62)	5.01 (3.42–7.35)
Frequency of listening to radio					
At least once a week	Ref.	Ref.	Ref.	Ref.	Ref.
Less than once a week	0.56 (0.31–1.02)	0.94 (0.62–1.43)	0.85 (0.40–1.84)	0.48 (0.24–0.94)	0.72 (0.24–2.16)
Not at all	1.07 (0.70–1.64)	1.96 (1.42–2.72)	2.98 (1.64–5.39)	2.51 (1.55–4.06)	7.83 (3.53–17.35)
Frequency of watching television					
At least once a week	Ref.	Ref.	Ref.	Ref.	Ref.
Less than once a week	1.04 (0.72–1.49)	1.02 (0.79–1.31)	1.34 (1.00–1.82)	1.10 (0.83–1.46)	1.17 (0.86–1.59)
Not at all	1.46 (1.15–1.86)	1.24 (1.05–1.47)	1.76 (1.44–2.16)	1.49 (1.23–1.80)	2.29 (1.87–2.80)
Husband/partner’s educational attainment					
Higher	Ref.	Ref.	Ref.	Ref.	Ref.
Complete secondary	3.91 (2.96–5.17)	4.24 (3.43–5.24)	8.05 (5.40–12.01)	8.91 (5.99–13.26)	12.62 (6.53–24.37)
Incomplete secondary	2.73 (2.17–3.45)	8.52 (7.35–9.87)	20.28 (15.10–27.23)	27.38 (20.24–37.03)	38.45 (22.53–65.61)
Complete primary	3.79 (2.55–5.62)	16.15 (12.38–21.07)	78.67 (54.45–113.65)	78.79 (54.16–114.64)	133.16 (74.42–238.28)
Incomplete primary	6.83 (4.36–10.69)	33.89 (24.23–47.40)	142.04 (93.18–216.52)	360.98 (237.02–549.77)	561.75 (306.38–1029.97)
No education	2.46 (0.93–6.51)	47.53 (26.56–85.04)	342.53 (181.87–645.10)	993.67 (528.89–1866.91)	4153.95 (1934.49–8919.81)

^1^Survey multinomial regression where survey weight for 2004 was used to estimate relative risk ratio and 95% Confidence Interval.

For mothers who did not listen to the radio at all, the probability of all levels of education increased from 2004–2018, although this was largest for no education (1.81 to 7.83) (Table 2). The probability for all levels of education decreased among women who watch television (Table 3). The probability of all levels of maternal education also decreased across all wealth index groups (Tables 2 and 3). If a woman partner had no education, then the likelihood of her education levels increased over time. Conversely, women’s likely education level appeared to decrease as their partner’s education level increased.

## Discussion

In Bangladesh, from 2004–2018, average maternal education level increased; accordingly, the proportion of women with no education decreased in this period. However, this study has identified that the prediction value of certain factors for different levels of women’s education has changed from 2004–2018. There was a significant increase in Bangladesh’s total population from 2004–2018 [23]. Parallel to this were initiatives implemented by Bangladeshi policymakers to meet the fundamental needs of the Bangladeshi population. One significant initiative was the introduction of Bangladesh’s first education policy in 2010, which established mandatory universal primary education (up to Grade 8) in all streams (general, madrassa and vocational) [13]. The policy is supported by practical assistance, including waiving of girls’ education fees, providing a stipend for girls up to Grade 12 and increasing supplies of basic education materials in schools. The policy also acknowledged the need to increase community and social awareness to motivate parents to send female children to school [13].

Media exposure is a factor in maternal education level; however, this study observed an overall mixed effect across maternal education levels. There has been a gradual shift in modern mass media from traditional paper and print to electronic media. Bangladesh is now the ninth-largest mobile market globally by unique subscribers [24]. As of January 2019, approximately 95.66% of Bangladeshi households have mobile phone or smartphone access, compared to 50% in 2010. The sustained efforts of the Bangladeshi Government to support country-wide digitalisation by ensuring secure, safe, stable and easy internet access have undoubtedly played a role in this. There is a need to empirically investigate the overall acceptance and consequences of this shift to electronic media, including its effects on maternal education levels.

Level of maternal education was similar regardless of residency (urban or rural). The most likely explanations for such similarity are overall infrastructure improvement, accessible and safe public transportation, enhancement of school facilities, appointment of suitably qualified teachers, and the availability and assurance of social security in rural areas [25, 26].

Paternal education is equally important as maternal education [27]. Husband’s/partner’s education level in relation to women’s education level in this study could be explained by males, even non-educated males, not marrying non-educated females. However, the large estimates with a very wide confidence interval may be due to very small sample sizes in different education groups. In any case, the estimates of associations for the complete secondary groups indicate the importance of both maternal and paternal education. This encourages further research to evaluate current educational strategies and the relative importance and influence of paternal education in maternal education, in Bangladesh and other countries.

Early marriage is a potential barrier to continued schooling and likely contributes to dropout [14]. At least one-third of the participating mothers were married by the age of 18 years or earlier. Given that women’s minimum age for marriage in Bangladesh is 18 years, such a high rate of underaged marriage is concerning and warrants additional efforts at the policy level. The probability of all maternal education levels decreased from 2004–2018 across all wealth index groups, indicating the overall increase in women’s education irrespective of economic class. Notably, maternal education levels decreased among the richest and richer wealth index groups and increased among mothers in the poorer and poorest wealth index groups. It is hard to draw any conclusions on this without further investigation to understand the complex socio-economic variable of wealth index. For example, the biggest change in maternal education levels was observed in the poorest wealth index group, but the absolute number of women in this group with some level of education (i.e., other than no education) is still very low compared to other wealth index groups. One could speculate that certain factors ensure delivery of education to mothers regardless of their economic class, or, more likely, mothers from low-income families in Bangladesh are being emphasised to receive proper education.

Bangladesh is now food independent, with its domestic agricultural output sufficient to ensure food security, thereby reducing maternal and child malnutrition and improving reproductive health [28]. Improved maternal education has been the most influential factor behind this achievement [2, 17]. Maternal education also contributes to the national economy, evidenced by the 72% gross increase in Bangladesh’s GDP from 2004 (USD475.29) to 2018 (USD 1698.35). The observed 67% gross decrease in no education level and similar gross increase in higher education level among women from 2004–2018 in Bangladesh may be correlated with this. Concerningly, Bangladesh does not have an education law to legislate the aforementioned 2010 education policy or protect citizens’ rights to education. Such legislation and reassessment of the current education policy are essential to address the many recent changes in the Bangladeshi education system (changed assessment system, introduction of parallel English-language curriculum, etc.) and changed global demands.

### Limitations and strengths

This study’s main strengths are the large, nationally representative sample and the 15-year study duration permitted by the BDHS using almost identical questionnaires and sampling schemes across the years. However, the data captured by the BDHS were not collected primarily to address the present study’s research aims. Thus, only a few factors potentially associated with maternal education were investigated in this study and further research is required.

## Conclusion

There was an overall increase in maternal education level in Bangladesh from 2004–2018. This improvement in maternal education level was equally observed in rural and urban areas and irrespective of economic class. However, paternal education is equally important, and the effects of this and the shift towards modern electronic devices on maternal education need to be further explored. Further, considering the demonstrated importance of women’s education in national progress and long-term sustainability, Bangladesh should consider legislating its citizens’ rights to education and certain aspects of the current education policy.

## Supporting information

S1 Table Descriptive statistics (unweighted proportion) of exposure variables among maternal education level groups between 2004 and 2018. (DOCX)

## Abbreviations

BDHS: Bangladesh Demographic Health Survey
LMICs: Low- and middle-income countries
MDGs: Millennium Development Goals
SDGs: Sustainable Develop Goals

## References

[1] Hamad R, Elser H, Tran DC, Rehkopf DH, Goodman SN. How and why studies disagree about the effects of education on health: A systematic review and meta-analysis of studies of compulsory schooling laws. Social Science & Medicine. 2018;212:168–78. 30036767 10.1016/j.socscimed.2018.07.016 PMC6209316

[2] Hasan MT, Soares Magalhaes RJ, Williams GM, Mamun AA. The role of maternal education in the 15‐year trajectory of malnutrition in children under 5 years of age in B angladesh. Maternal & child nutrition. 2016;12(4):929–39. 25720451 10.1111/mcn.12178 PMC6860139

[3] Güneş PM. The role of maternal education in child health: Evidence from a compulsory schooling law. Economics of Education Review. 2015;47:1–16.

[4] Carneiro P, Meghir C, Parey M. Maternal education, home environments, and the development of children and adolescents. Journal of the European Economic Association. 2013;11(suppl_1):123–60.

[5] Bado AR, Sathiya Susuman A. Women’s education and health inequalities in under-five mortality in selected sub-Saharan African countries, 1990–2015. Plos one. 2016;11(7):e0159186. doi: 10.1371/journal.pone.0159186 27442118 PMC4956109

[6] Dubow EF, Boxer P, Huesmann LR. Long-term effects of parents’ education on children’s educational and occupational success: Mediation by family interactions, child aggression, and teenage aspirations. Merrill-Palmer quarterly (Wayne State University Press). 2009;55(3):224. 20390050 10.1353/mpq.0.0030 PMC2853053

[7] Forshaw J, Gerver SM, Gill M, Cooper E, Manikam L, Ward H. The global effect of maternal education on complete childhood vaccination: a systematic review and meta-analysis. BMC infectious diseases. 2017;17(1):1–16. doi: 10.1186/s12879-016-2122-x 29281990 PMC5745980

[8] Lundborg P, Nilsson A, Rooth D-O. Parental education and offspring outcomes: evidence from the Swedish compulsory School Reform. American Economic Journal: Applied Economics. 2014;6(1):253–78.

[9] Mensch BS, Chuang EK, Melnikas AJ, Psaki SR. Evidence for causal links between education and maternal and child health: systematic review. Tropical Medicine & International Health. 2019;24(5):504–22. doi: 10.1111/tmi.13218 30767343 PMC6519047

[10] Psaki SR, Mensch BS, Chuang E, Melnikas AJ. Evidence for Causal Links Between Education and Maternal and Child Health, Sexual and Reproductive Health, and Malaria: A Systematic Review. Population Association of America Denver, Colorado: Population Council. 2018.

[11] Vikram K, Vanneman R. Maternal education and the multidimensionality of child health outcomes in India. Journal of biosocial science. 2020;52(1):57. doi: 10.1017/S0021932019000245 31112112 PMC7068132

[12] Jackson MI, Kiernan K, McLanahan S. Maternal education, changing family circumstances, and children’s skill development in the United States and UK. The Annals of the American Academy of Political and Social Science. 2017;674(1):59–84. doi: 10.1177/0002716217729471 29563643 PMC5857959

[13] National Education Policy 2010. Ministry of Education. Government of the People’s Republic of Bangladesh. (accessed date: 28 Jan 2021). https://reliefweb.int/sites/reliefweb.int/files/resources/02.National-Education-Policy-2010-English.pdf.

[14] Global Education Monitoring Report 2019 –Gender Report: Building bridges for gender equality. Paris, UNESCO. (accessed date: 28 Jan 2021). https://unesdoc.unesco.org/ark:/48223/pf0000368753.

[15] Altbach PG, Reisberg L, Rumbley LE. Trends in global higher education: Tracking an academic revolution. A Report Prepared for the UNESCO 2009 World Conference on Higher Education. 2009. (accessed date: 28 Jan 2021) http://atepie.cep.edu.rs/public/Altbach,_Reisberg,_Rumbley_Tracking_an_Academic_Revolution,_UNESCO_2009.pdf. Boston College Center for International Higher Education Chestnut Hill, MA.

[16] Damon A, Glewwe P, Wisniewski S, Sun B. Education in Developing Countries-what Policies and Programmes Affect Learning and Time in School? Expertgruppen för biståndsanalys (EBA), 2016 9188143120.

[17] Hasan MT, Magalhaes RJS, Williams GM, Mamun AA. Forecasting the progress towards the target of Millennium Development Goal 1C in children under 5 years of age in Bangladesh. Public health nutrition. 2015;18(10):1728–36. doi: 10.1017/S1368980014003279 25648950 PMC10271520

[18] Kamal SM. Maternal education as a determinant of neonatal mortality in Bangladesh. Journal of Health Management. 2012;14(3):269–81.

[19] Parvazian S, Gill J, Chiera B. Higher education, women, and sociocultural change: A closer look at the statistics. Sage Open. 2017;7(2):2158244017700230.

[20] Mitra SN, Ali MN, Islam S, Cross AR, Saha T. Bangladesh Demographic and Health Survey. 1993–1994. Calverton, Maryland: National Institute of Population Research and Training (NIPORT), Mitra and Associates, and Macro International Inc. (accessed date: 28 Jan 2021). https://dhsprogram.com/pubs/pdf/FR60/FR60.pdf.

[21] National Institute of Population Research and Training (NIPORT), and ICF. 2019. Bangladesh Demographic and Health Survey 2017–18: Key Indicators. Dhaka, Bangladesh, and Rockville, Maryland, USA: NIPORT, and ICF. (accessed date: 28 Jan 2021). https://dhsprogram.com/pubs/pdf/PR104/PR104.pdf.

[22] Demographic and Health Survey. The DHS Program. USAID. https://dhsprogram.com/Countries/Country-Main.cfm?ctry_id=1

[23] Population Projection of Bangladesh: Dynamics and trend 2011–2061. November 2015. Bangladesh Bureau of Statistics (BBS), Statistics and Informatics Division (SID), Ministry of Planning, Government of The People’s Republic of Bangladesh. (assessed on 28 Jan 2021). http://203.112.218.65:8008/WebTestApplication/userfiles/Image/PopMonographs/PopulationProjection.pdf.

[24] Country overview: Bangladesh. Mobile industry driving growth and enabling digital inclusion. GSMA Intelligence. 2018. (accessed date: 28 Jan 2021). https://data.gsmaintelligence.com/api-web/v2/research-file-download?id=30933394&file=Country%20overview%20Bangladesh.pdf.

[25] The developement vision and poverty reduction framework. Steps Towards Change. National Strategy for Accelerated Poverty Reduction II, FY 2009–11. October 2012. International Monetary Fund. Washington, D.C. (accessed date: 14 July 2021) https://www.elibrary.imf.org/view/journals/002/2012/293/002.2012.issue-293-en.xml.

[26] Innovative Strategies for accelerated human resource development In South Asia Teacher Professional Development. Special focus on Bangladesh, Nepal, and Sri Lanka. 2017. Asian Development Bank, Philippines (accessed date: 14 July 2021) https://www.adb.org/sites/default/files/publication/385091/teacher-professional-development-sa.pdf.

[27] Smith TK, Duggan A, Bair‐Merritt MH, Cox G. Systematic review of fathers’ involvement in programmes for the primary prevention of child maltreatment. Child Abuse Review. 2012;21(4):237–54

[28] Osmani S, Ahmed A, Ahmed A, Hossain N, Huq S, Shahan A. Strategic Review of Food Security and Nutrition in Bangladesh. World Food Programme. September 2016. (accessed date: 28 Feb 2021) https://cdn.wfp.org/wfp.org/publications/Bangladesh_Strategic_Review_full_report.pdf?_ga=2.217816981.24139821.1611824927–1964655570.1611824927.

