# Supplementary material for: Trend of maternal education in Bangladesh from 2004–2018: Analysis of demographic surveillance data
Source: PLoS One. 2022 Jan 31;17(1):e0255845. doi: 10.1371/journal.pone.0255845 (PMC8803158; doi:10.1371/journal.pone.0255845)
Supplement: S1 Table — (DOCX) [file pone.0255845.s001.docx]

**S1 Table**: Descriptive statistics (unweighted proportion) of exposure variables among maternal education level groups between 2004 and 2018

| Exposure variables | 2017-18 (%) | | | | | |  | 2004 (%) | | | | | | |
| --- | --- | --- | --- | --- | --- | --- | --- | --- | --- | --- | --- | --- | --- | --- |
|  | Higher  (N=2730) | Complete secondary  (N=822) | Incomplete secondary  (N=6608) | Complete primary (N=2009) | Incomplete primary  (N=3891) | No education  (N=2790) |  | Higher  (N=690) | Complete secondary  (N=301) | Incomplete secondary  (N=2644) | Complete primary  (N=1050) | Incomplete primary  (N=2320) | No education  (N=4412) |  |
| Area |  |  |  |  |  |  |  |  |  |  |  |  |  |  |
| Urban | 44.47 | 53.53 | 65.51 | 67.5 | 68.98 | 70.18 |  | 35.8 | 44.19 | 61.76 | 69.14 | 68.71 | 72.26 |  |
| Rural | 55.53 | 46.47 | 34.49 | 32.5 | 31.02 | 29.82 |  | 64.2 | 55.81 | 38.24 | 30.86 | 31.29 | 27.74 |  |
| Religion |  |  |  |  |  |  |  |  |  |  |  |  |  |  |
| Islam | 87.55 | 90.02 | 89.65 | 91.34 | 91.6 | 90.25 |  | 84.93 | 83.72 | 87.56 | 91.43 | 89.87 | 89.96 |  |
| Non-Islam | 12.45 | 9.98 | 10.35 | 8.66 | 8.4 | 9.75 |  | 15.07 | 16.28 | 12.44 | 8.57 | 10.13 | 10.04 |  |
| Wealth index |  |  |  |  |  |  |  |  |  |  |  |  |  |  |
| Richest | 57 | 44.04 | 22.17 | 13.34 | 9.46 | 7.53 |  | 76.81 | 65.78 | 38.73 | 26.38 | 16.08 | 11.36 |  |
| Richer | 22.12 | 25.3 | 23.06 | 21.01 | 17.12 | 15.13 |  | 15.22 | 21.59 | 26.82 | 28.38 | 19.87 | 14.42 |  |
| Middle | 12.2 | 15.21 | 22.47 | 20.31 | 20.23 | 18.14 |  | 6.09 | 7.97 | 18.84 | 19.9 | 22.76 | 19.11 |  |
| Poorer | 5.75 | 9.49 | 18.34 | 22.85 | 24.8 | 25.84 |  | 1.59 | 3.99 | 11.12 | 15.43 | 22.46 | 23.89 |  |
| Poorest | 2.93 | 5.96 | 13.95 | 22.5 | 28.4 | 33.37 |  | 0.29 | 0.66 | 4.5 | 9.9 | 18.84 | 31.23 |  |
| Frequency of listening to radio | |  |  |  |  |  |  |  |  |  |  |  |  |  |
| At least once a week | 5.6 | 4.14 | 2.04 | 1 | 1.08 | 0.36 |  | 41.45 | 47.51 | 44.33 | 38 | 33.58 | 23.91 |  |
| Less than once a week | 8.32 | 4.01 | 3.07 | 1.54 | 0.85 | 0.32 |  | 11.59 | 12.29 | 11.72 | 13.05 | 11.94 | 10.31 |  |
| Not at all | 86.08 | 91.85 | 94.88 | 97.46 | 98.07 | 99.32 |  | 46.96 | 40.2 | 43.95 | 48.95 | 54.48 | 65.78 |  |
| Frequency of watching television | |  |  |  |  |  |  |  |  |  |  |  |  |  |
| At least once a week | 78.68 | 68.49 | 59.79 | 45.74 | 45.28 | 35.77 |  | 85.51 | 78.74 | 64.67 | 51.33 | 43.19 | 31.6 |  |
| Less than once a week | 6.01 | 7.42 | 8.84 | 10.35 | 9.79 | 8.42 |  | 3.48 | 5.65 | 8.51 | 11.43 | 10 | 9.16 |  |
| Not at all | 15.31 | 24.09 | 31.37 | 43.9 | 44.92 | 55.81 |  | 11.01 | 15.61 | 26.82 | 37.24 | 46.81 | 59.25 |  |
| Frequency of reading newspaper | | |  |  |  |  |  |  |  |  |  |  |  |  |
| At least once a week | 20.7 | 7.91 | 1.91 | 0.35 | 0.05 | 0.04 |  | 55.8 | 37.87 | 14.83 | 4.29 | 0.69 | 0.02 |  |
| Less than once a week | 22.05 | 13.99 | 7.16 | 1.99 | 0.67 | 0.04 |  | 21.59 | 33.22 | 23.87 | 16.38 | 3.19 | 0.07 |  |
| Not at all | 57.25 | 78.1 | 90.94 | 97.66 | 99.28 | 99.93 |  | 22.61 | 28.9 | 61.31 | 79.33 | 96.12 | 99.91 |  |
| Husband/partner's educational attainment | |  |  |  |  |  |  |  |  |  |  |  |  |  |
| Higher | 71.5 | 40.02 | 13.98 | 4.08 | 1.75 | 0.65 |  | 83.19 | 60.13 | 19.55 | 7.52 | 2.72 | 0.93 |  |
| Complete secondary | 7.33 | 17.64 | 7.13 | 3.63 | 2.08 | 1.18 |  | 4.93 | 20.93 | 9.34 | 6.29 | 3.02 | 1.61 |  |
| Incomplete secondary | 15.86 | 26.64 | 36.86 | 26.13 | 17.63 | 9.89 |  | 8.84 | 13.29 | 37.93 | 27.43 | 22.28 | 10.97 |  |
| Complete primary | 3.04 | 7.42 | 15.13 | 21.85 | 12.44 | 8.1 |  | 1.45 | 1.99 | 9.46 | 20.95 | 9.83 | 7.37 |  |
| Incomplete primary | 1.76 | 7.18 | 17.98 | 23.79 | 32.77 | 20.86 |  | 0.72 | 2.33 | 12.18 | 15.9 | 27.11 | 16.5 |  |
| No education | 0.51 | 1.09 | 8.91 | 20.51 | 33.33 | 59.32 |  | 0.87 | 1.33 | 11.54 | 21.9 | 35.04 | 62.62 |  |
